# Protein Homeostasis, Aging and Alzheimer’s Disease

**DOI:** 10.1007/s12035-012-8246-0

**Published:** 2012-02-24

**Authors:** Tobias Morawe, Christof Hiebel, Andreas Kern, Christian Behl

**Affiliations:** Institute for Pathobiochemistry, University Medical Center of the Johannes Gutenberg University, Duesbergweg 6, 55099 Mainz, Germany

**Keywords:** Alzheimer’s disease, Proteostasis, Aging, Chaperones, UPS, Autophagy

## Abstract

Alzheimer’s disease (AD) is one key medical challenge of the aging society and despite a great amount of effort and a huge collection of acquired data on molecular mechanisms that are associated with the onset and progression of this devastating disorder, no causal therapy is in sight. The two main hypotheses of AD, the amyloid cascade hypothesis and the Tau hypothesis, are still in the focus of AD research. With aging as the accepted main risk factor of the most important non familial and late onset sporadic forms of AD, it is now mandatory to discuss more intensively aspects of cellular aging and aging biochemistry and its impact on neurodegeneration. Since aging is accompanied by changes in cellular protein homeostasis and an increasing demand for protein degradation, aspects of protein folding, misfolding, refolding and, importantly, protein degradation need to be linked to AD pathogenesis. This is the purpose of this short review.

## Introduction

Alzheimer’s disease (AD) is a progressive neurodegenerative disorder starting with mild memory loss and manifesting in severe cognitive decline. Neuropathologically, the disease is characterized by an extensive deposition of Amyloid β (Aβ) peptides in extracellular amyloid plaques and by intracellular neurofibrillary tangles (NFTs) of hyper-phosphorylated Tau protein [1]. Sporadic late-onset AD is the most common form of dementia in the elderly and is strongly associated with age. In rare cases (<5%), AD is inherited and results in an early disease onset. One genetic risk factor for late-onset AD that has been reported throughout many studies is Apolipoprotein E (ApoE) which physiologically functions as a ligand in receptor-mediated endocytosis of lipoprotein particles [2, 3]. Several single nucleotide polymorphisms in *apoE* lead to alterations in the coding sequence and result in three common isoforms called *apoE2*, *apoE3* and *apoE4* with the *E4* allele being an AD risk factor and *E2* being protective [4–6]. Strong evidence suggests that ApoE influences AD via its effect on Aβ metabolism; however, the details of this process have to be fully elucidated. Positional cloning studies of familial AD (FAD) cases have identified mutations in three genes, *amyloid precursor protein* (*app*), *presenilin 1* and *2* (*ps1*, *ps2*), which are tightly linked to the generation of Aβ peptides [7]. Proteolytic processing of amyloid precursor protein (APP) by BACE (β-site APP cleaving enzyme) is followed by cleavage by γ-secretase containing presenilins and results in the release of Aβ peptides of different length. Aβ40 and Aβ42 (40 or 42 amino acids, respectively) are the most prominent Aβ peptides and since the C-terminus has important implication for toxicity, the longer Aβ42 peptide exhibits stronger neurotoxic properties. The FAD-linked mutations cause an elevated production of amyloidogenic Aβ, which is the basis for the amyloid hypothesis of AD, stating that Aβ peptides are the key-players in neuronal dysfunction and subsequent neurodegeneration in AD [8]. Recently, it has been suggested that the soluble oligomeric forms of Aβ42 are the potentially harmful species and that these oligomers affect various important cellular mechanisms, resulting in decreased functionality and survival of neuronal cells [9]. However, Aβ peptides are not exclusively generated under pathological conditions as they are part of the normal cellular metabolism and a significant load of amyloid plaques is also observed in brains of healthy aged individuals [10, 11]. This challenges the amyloid hypothesis of AD and up to date the nature of the disease-relevant alteration in aged neurons remains unclear.

Although Alois Alzheimer already described protein deposits in the brain of demented patients, the question why distinct proteins that are potentially causative for the disease accumulate in discrete brain regions in the course of aging remains unanswered. Today, we know that the progressive deposition of misfolded and aggregation-prone proteins in defined regions of the nervous system is not unique to AD, but a well-described characteristic of several neurodegenerative disorders, including Parkinson’s disease (PD), Huntington’s disease (HD) and Amyotrophic Lateral Sclerosis (ALS) [12]. Recently, it has been proposed that the accumulation of disease-relevant proteins in aggregates might actually even be considered as a protective mechanism to dispose these proteins and remove them from the cellular metabolism [13, 14]. Generally, within the crowded cellular environment, proteins are always at risk for denaturation. Changing genetic and environmental factors, as during pathology or aging, challenge the integrity of the cellular protein homeostasis (proteostasis). Therefore, the stability and metabolism of the proteome needs to be controlled carefully and is carried out by a complex network of several hundred evolutionarily conserved proteins. This network, including molecular chaperones, the ubiquitin–proteasome system (UPS) and the autophagy system is stringently regulated and indispensable for the maintenance of proteostasis. All main players of this network are ubiquitously expressed and consequently also present in neuronal tissue.

Molecular chaperones, most be prominently represented by the heat shock protein 70 (HSP70) family and their regulators, are responsible for correct folding and refolding of proteins as well as for the transport of misfolded proteins to the protein-degradation systems [15]. The chaperone network is controlled by the activity of transcription factors, e.g. heat shock transcription factor 1 (HSF1), which rapidly adjusts the cellular chaperone capacity during stress conditions. The UPS is the major protein-degradation machinery of the cell and controls the metabolism of cytosolic proteins and the degradation of misfolded proteins. Proteins that are destined for proteasomal degradation become specifically tagged with the protein ubiquitin in a well-organized enzymatic cascade [16]. Interestingly, also a ubiquitin-independent way of proteasomal degradation has been described recently that mostly involves small and misfolded proteins [17]. As an additional mechanism for protein degradation the term autophagy summarizes degradation pathways that deliver their substrates to the lysosome [18]. The major autophagic systems described so far are macroautophagy and chaperone-mediated autophagy (CMA). Macroautophagy is a complex process that involves the formation of a membrane structure, the autophagosome, which engulfs bulk cellular material (proteins and organelles) and subsequently fuses with the lysosome. CMA involves the constitutively expressed heat shock cognate 70 (HSC70) that targets client proteins with a specific consensus motif directly to the lysosome [19]. In fact, HSP70 is also an important factor to assure selectivity of macroautophagy of degradation-prone proteins in a distinct pathway, mediated via the co-chaperone BCL2-associated athanogene 3 (BAG3) [20].

Aging and disease challenge the proteostasis network and the accumulation of instable proteins results in wide-spread protein aggregation [21, 22]. Our discussion presented here attempts to integrate two major factors that are associated with neurodegeneration, protein homeostasis and aging. Most importantly, we focus on the role of specific pathways which are needed to restore and maintain proteostasis during aging and their interplay with a special focus on impaired proteostasis in AD, which may add to a novel view and understanding of AD pathogenesis. Since recently many excellent reviews on various aspects providing in depth discussions have been presented, we are concentrating here on the integration of these different aspects. Therefore, we frequently refer to review work with the recommendation of further reading rather than trying to fully cover the discussed issues by their original publications.

## Maintenance of Proteostasis via Molecular Chaperones: the First Line of Defense Against Protein Misfolding

Cells permanently encounter the problem to maintain the integrity and functionality of the proteome. Within the crowded cellular environment, the correct conformation of proteins must be controlled and misfolded and irreversibly damaged proteins must be efficiently refolded or removed. Central players of the protein homeostasis system are molecular chaperones that sense misfolded proteins and, when refolding fails, direct them to the protein-degradation pathways. Molecular chaperones are specified as proteins that interact with and participate in folding or refolding of non-native proteins. Therefore, chaperones help unfolded proteins to achieve their functional conformation without being present in the final structure [15, 23–27]. They exert a multitude of activities, including de novo folding, refolding of denatured proteins, transport to subcellular compartments, oligomeric assembly and disposal by proteolytic degradation [28, 29]. Different classes of chaperones work together to form co-operative networks and are often termed heat shock proteins (HSPs), because they are sensitively up-regulated under stress conditions in which the amount of misfolded proteins is increased [30]. Chaperones, which are involved in protein folding and refolding, such as HSP70, HSP90 and chaperonin (HSP60), promote folding activity through ATP/cofactor-regulated binding and release cycles [15, 31]. They recognize short hydrophobic amino acid stretches of misfolded proteins and can co-operate with ATP-independent chaperones, e.g. small HSPs (sHSPs), to prevent protein aggregation.

HSP70 proteins are central players in proteostasis control and increasing HSP70 levels prevent protein aggregation in various disease models that are based on the expression of aggregation-prone proteins [32, 33]. The activity of HSP70 is ATP dependent and is controlled by chaperones of the HSP40 family as well as nucleotide exchange factors (NEFs) [34, 35]. After ATP hydrolysis, a NEF binds to the ATPase domain of HSP70 and catalyzes the nucleotide exchange, which results in substrate release. Thus, chaperone binding to the hydrophobic region of misfolded proteins transiently blocks aggregation and the subsequent ATP-dependent release allows controlled folding of the client protein. HSP40/DNAJs, HSC70-interacting protein (HIP), BCL2-associated athanogene 1 (BAG1) and BAG3 are prominent co-chaperones that control the ATP-dependent activity of HSP70. DNAJ proteins generally enhance the ATPase activity of HSP70, but their action can lead to different fates of their bound substrates. DNAJB1 is an example for a DNAJ protein that supports substrate folding, whereas DNAJB2-bound clients are degraded by the proteasome [36]. The co-chaperone HIP stabilizes the ADP-bound state of HSP70 and co-operates with HSP70 in protein folding. BAG1 inhibits the HSP70 chaperone activity, competing with the stimulatory action of HIP [37]. Other HSP70-binding cofactors are the ubiquitin ligases carboxy terminus of HSC70-interacting protein (CHIP) and PARKIN, which provide a link between HSP70, co-chaperones and the UPS, resulting in client ubiquitination and degradation by the proteasome [34, 38, 39]. Recently, it has been shown that BAG3 interacts with HSP70 and directs the chaperone and its substrates into the autophagic pathway, providing an association of HSP70, co-chaperones and autophagy [40]. An extensive description of the HSP70 machinery, the exact structure-function relationship between HSP70 and its various cofactors, has been excellently reviewed elsewhere [34].

The functional variability of the chaperone system that is exerted by a multitude of single proteins highlights the complexity of this proteostasis network. However, limited knowledge exists on the exact composition and capacity of the chaperone system within a cell or a certain tissue. A general view assumes that total levels of cellular chaperones exceed the actual requirements and that a sufficient amount of chaperones is free of clients. Thus, the network has excess capacity to initially deal with sudden additional chaperone requirements, as exhibited during stress conditions [41]. An opposite view proposes that the capacity of the chaperone network is always closely titrated to the actual demand of chaperone activity and that the system is otherwise rapidly adapted to conditions of increased requirements [41]. The analysis of the chaperone capacity of neuronal and muscular tissue of *Caenorhabditis elegans* (*C. elegans*) demonstrates that these tissues indeed display different folding activities and that neurons are particularly sensitive to protein denaturation during heat stress [42]. However, the adaptation of the chaperone network during aging is critical as it is well acknowledged that protein aggregation and disruption of proteostasis are characteristic for aged cells [21]. Several studies have clearly implicated a role of chaperones in aging. The periodical application of mild heat stress decreases mortality in *Drosophila melanogaster* and *C. elegans*, which is mediated by HSP70 activity and the overexpression of HSP70 in *C. elegans* increases the life span of the nematode [43–45]. Several reports have analyzed chaperone protein or mRNA levels in aged cells and found increased or basal amounts, whereas the stress-mediated induction of chaperone expression is impaired. The transcription of chaperone genes in response to stress conditions is controlled by the transcription factor HSF1, which shows an impaired DNA-binding potential in aged cells [46]. A reduced activity of HSF1 in *C. elegans* results in a shortened life span and, conversely, the enhanced expression of the transcription factor increases the life span. HSF1 activity is also essential for the extended life span of the extremely long-lived daf-2/Insulin/IGF-1 receptor mutants of *C. elegans* [47, 48]. Thus, HSF1 and chaperone activity can promote longevity, demonstrating a clear association of chaperones, proteostasis and aging.

## Molecular Chaperones Get in Touch with the Protein Degradation Machineries

Some of the factors mentioned so far are involved in linking chaperone functions with cellular protein-degradation pathways, the UPS and autophagy, for the removal of misfolded proteins. Besides protein aggregation, one factor that induces ubiquitination is protein damage caused by free radical oxygen species (ROS) and oxidative stress. Most likely, irreversible oxidation may activate chaperones and the UPS to induce protein repair of misfolded proteins and lead to ubiquitination and protein degradation. During aging, mitochondria are affected and produce increasing amounts of ROS. In particular, the mitochondrial respiratory chain is strongly linked to the production of ROS and as one consequence may cause protein dysfunction, apoptosis, necrosis, aging and disease [49, 50]. Protein oxidation leads to a change in protein conformation and function and chaperones may sense such changes and in turn activate the UPS as a quality control system. Depending on the degree of oxidation, irreversible oxidation and loss of protein function may lead to degradation and/or accumulation of damaged proteins and to the formation of so-called aggresomes, which display a high autophagic activity [51, 52].

The UPS is a complex enzymatic pathway that starts with the ligation of ubiquitin, a 76-amino-acid-long and highly conserved protein, to other cellular proteins and thus labels them for degradation. This process consists of three steps. Initially the C-terminal end of ubiquitin is activated by ATP-dependent phosphorylation and formation of a thiol ester via an activating enzyme, E1. It is then transferred to a thiol group of an ubiquitin-carrier protein, E2. The E3 ligase directs ubiquitin from E2 to an ε-amino group of the target protein [53, 54]. The C-terminus of an additional ubiquitin protein can be ligated onto one of the seven lysine residues within the attached ubiquitin molecule. For degradation via the proteasome, target proteins need to be polyubiquitinated. Ubiquitin–ubiquitin linkages between either the C-terminus and lysine residues K48 or K63 are the major recognition signals for proteasomal degradation. Ubiquitin chains also occur among other lysine residues, whereas ubiquitin extension via K6 is associated with DNA repair, K11 with endoplasmatic reticulum-associated protein degradation and cell cycle regulation, K27 with ubiquitin fusion degradation, K29 with lysosomal degradation and K33 with kinase modification [55]. Monoubiquitination can modify the activity of the protein transport machinery and when attached to transmembrane proteins can serve as a sorting signal to direct their movement between different cellular compartments [56–59]. The polyubiquitinated proteins destined for degradation are processed by the multienzymatic proteasome complex: first they become deubiquitinated and then degraded by the 26S proteasome, a system that is composed of various proteasome subunits. The eukaryotic 20S proteasome core complex consists of four heptameric rings comprising two classes of seven non-identical but homologous subunits. The outer rings contain alpha-type subunits with gating function for substrate entrance and product release, while the beta-subunits exhibit peptide hydrolyzing activity. The 19S regulatory complex binds to the 20S catalytic core in a flexible manner and consists of six ATPases, forming a ring at the entrance of the core and exerting chaperone-like activity [60].

The UPS is the major degradation system in the cell for the degradation of short-lived, misfolded and defective proteins. An accumulation of polyubiquitinated proteins is reported for numerous neurological disorders [61], which suggests that UPS dysfunction plays a prominent role in the pathogenesis of neurodegenerative diseases and moreover in aging [62]. Since proteasomal activity decreases during aging over all, the degradation demand of the cell increases, which results in an age-associated induction of the autophagy pathway that delivers substrates for degradation ultimately to lysosomes [20, 63].

Autophagy is negatively regulated via the evolutionarily conserved enzymatic mammalian target of rapamycin complex 1 (mTORC1). In turn, if mTOR activity is inhibited by rapamycin, the autophagy pathway is switched on. Rapamycin exerts its stimulatory effect on autophagy by preventing mTOR phosphorylation at Ser-2448 and thereby subsequently blocks mTOR signaling [64]. So far, many different autophagic systems have been described including macroautophagy and CMA that form the main pathways. Macroautophagy is a multi-step process by which cytosolic material is sequestered into a double-layered membrane structure, the autophagosome, and is delivered to the lysosome for degradation. It is a highly orchestrated process and involves autophagy-related (ATG) proteins. More than 30 genes have been identified in genetic analyses of yeast mutants which have defects in autophagic function [65]. After autophagy stimulation through mTOR inhibition, the formation of phagophores (autophagosome precursors/pre-autophagosomal structures) is initiated in a not yet completely understood mechanism. A subset of ATG proteins is required for autophagosome formation: First, the ATG9 system including ATG9, the ATG1 kinase complex (ATG1 and ATG13), ATG2 and ATG18; second, the phosphatidylinositol 3-OH kinase (PI(3)K) complex consisting of vacuolar protein sorting (VPS)34, VPS15, BECLIN 1/ATG6 and ATG14 and third, the ubiquitin-like protein (Ubl) system composed of two Ubl proteins (LC3/ATG8 and ATG12), an E1 enzyme (ATG7), two analogues of E2 ubiquitin-conjugating enzymes (ATG10 and ATG3), an LC3/ATG8-modifying protease (ATG4), the protein target of ATG12 attachment (ATG5) and ATG16 [66]. BECLIN 1/ATG6 regulates autophagic trafficking and membrane trafficking in a variety of physiological and pathological processes [67]. Microtubule-associated protein 1 light chain 3 (LC3/ATG8) is commonly used as a marker for autophagic activity. With its E1-like activity, ATG7 converts cytosolic LC3-I to the membrane-associated LC3-II, which then is conjugated to phosphatidylethanolamine. LC3-II binds the inner and outer membrane of the forming autophagosome and provides a physical link between the autophagosome and LC3 interacting proteins, such as Sequestosome 1 (p62/SQSTM1) or Neighbor of brca1 gene 1 (NBR1). These so-called autophagy receptors can simultaneously bind to ubiquitinated proteins and LC3, employing their LC3-interacting motif and ubiquitin-associated domain. The identification of autophagy receptors, such as p62/SQSTM1 and NBR1 has provided a molecular link between ubiquitination and autophagy. Thus, p62/SQSTM1 and NBR1 are possibly also important bridging factors between UPS and autophagy [68]. Through self-oligomerization, which is stimulated by ubiquitin binding, p62/SQSTM1 sequesters ubiquitinated substrates in form of inclusion bodies. These inclusions are then specifically engulfed by the autophagosome membrane by recruiting LC3 [69, 70].

While macroautophagy is considered as a rather unspecific robust degradation process, CMA is a highly selective lysosomal pathway that removes a distinct subset of proteins containing a pentapeptide lysosome-targeting motif. These substrates can on the one hand directly be translocated into the lysosome after docking to the lysosomal receptor lysosomal-associated membrane protein 2A or on the other hand unfolded by a chaperone complex containing HSC70 and the co-chaperones BAG1, HIP, HSP70–HSP90 organizing protein (HOP) and HSP40/DNAJB1 [19]. These co-chaperones provide a direct link to the UPS and the folding and refolding activity of molecular chaperones. In the course of aging, a variety of proteins tend to aggregate and impair the UPS directly [71]. Therefore, these proteins, most of which are ubiquitinated, cannot be handled by the proteasome and have to be degraded by different protein clearance pathways. Recently, it has been shown that the cellular protein quality control (PQC) of polyubiquitinated proteins by proteasomal and autophagic systems is regulated by the HSP70 co-chaperones BAG1 and BAG3, respectively [40]. Proteasome activity decreases in an age-dependent manner in a cell model of replicative senescent human fibroblasts and is associated with an increased autophagic activity in aged cells. This correlates with decreased levels of BAG1 and increased levels of BAG3 in aged cells, an age-related BAG1/BAG3 switch that serves as an induction control of the macroautophagic pathway (BAG3-mediated selective macroautophagy) and may be considered as a backup of the UPS in PQC ([40, 72]). Therefore, the expression shift from BAG1 to BAG3 during aging, but also upon acute stress (e.g. proteasome inhibition, oxidative stress), can be considered as a physiologically important adaptive response [20, 40]. The BAG3-mediated selective pathways have been shown to be involved in a variety of disease causing processes. In a model for HD, BAG3 in concert with HSPB8 facilitates the disposal of polyQ43-Huntingtin by stimulating macroautophagy and this complex is also involved in Z-disc maintenance in flies, mice and men [73, 74]. Additionally, the transport of target proteins to the aggresome, a compartment with high autophagic activity, has been shown to be mediated by BAG3. This aggresome-targeting pathway involving BAG3 and HSP70 is distinct from other before-described mechanisms as it does not depend on substrate ubiquitination [75].

**Fig. 1:**
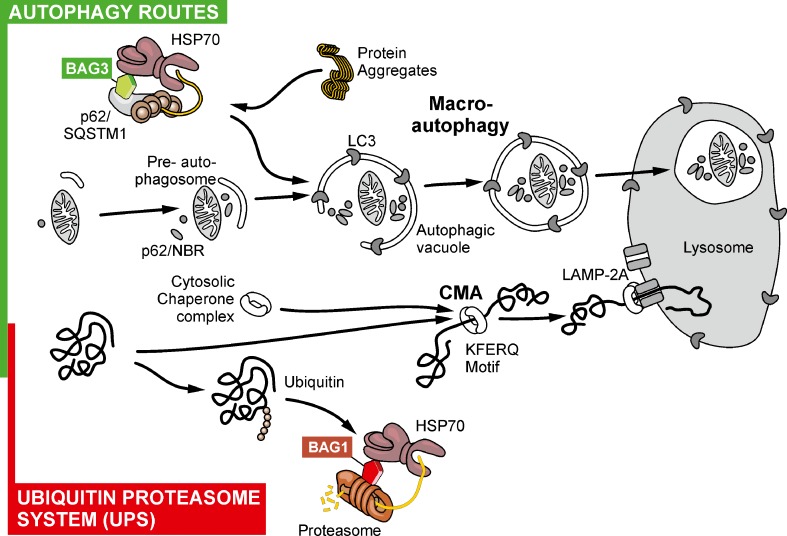
The ubiquitin–proteasome system (UPS) and various autophagy routes as the main pathways for protein degradation (regular turnover) and of misfolded and aggregated proteins (and mitochondria)

Taken together the molecular chaperone machinery, the UPS and the autophagy system are involved in PQC and maintaining proteostasis within aging and disease to prevent protein misfolding and aggregation. A summary of the main routes for protein degradation is shown in Fig. 1. As aggregated proteins are found in AD patients and aging is a prevailing risk factor for AD it is important to further characterize how these pathways are regulated and how misregulation possibly contributes to the pathology of AD.

## Proteostasis and AD

AD is characterized by the accumulation of intracellular Aβ (oligomers), extracellular high molecular weight deposits of Aβ peptides (fibrilliar forms), and the intracellular aggregation of hyper-phosphorylated Tau. Indeed, modification of the cellular proteostasis and the metabolism of Aβ and Tau have been proven to cause neuronal dysfunction and cell death [12]. The malfunctioning proteostasis control results in the accumulation of misfolded and aggregated proteins, which is a general hallmark of aging and neurodegeneration, as observed in PD, HD, ALS and AD. Aging in particular is characterized by decreasing proteostasis capacity and increasing protein damage which are in combination a major challenge for the cell due to the inability to maintain metastable proteins in folded states [76]. To counteract a cascade of protein destabilization caused by metastable proteins which escaped the PQC the proteostasis network including molecular chaperones, the UPS and autophagic pathways must manage the increasing burden of protein misfolding to maintain proteome stability [77]. Since aging is the major risk factor of AD [78–80], the age-dependent loss of proteostasis might be an important contributor to the pathology of AD. An overview of proteins that affect proteostasis in AD is shown in Table 1.

**Table 1:** Proteins affecting proteostasis in Alzheimer’s disease

Protein	Function	Reference
Aβ40/42	Decreases proteasome activity, modulates autophagy through mTOR signaling	[99–102, 104, 143–146]
Tau	Inhibitory effect of Tau aggregates on proteasome activity	[115]
HSF1	Induces APP gene during stress	[81, 82]
GRP78 (ER isoform of HSP70)	Modulates APP maturation and reduces Aβ40/42 secretion	[84, 85]
HSPs (HSP22, 27, 70, 90)	Small HSPs (HSP22, HSP27) bind to fibrillar amyloid plaques and inhibit their fibrillarisation; HSP70, HSP90 inhibit early stages of amyloid aggregation	[88, 89]
CHIP	E3 enzyme for phosphorylated Tau	[93, 117]
BAG1	Regulates proteasomal degradation of Tau together with HSP70	[97]
PS1	Increases production of Aβ42, essential for lysosomal proteolysis and autophagy by enabling the acidification of lysosomes required for protease activation	[163, 165]
UBB+1	Potently inhibits the degradation of polyubiquitinated substrates and therefore induces neuronal cell death	[120, 121]
UCH-L1	DUB needed for proteasomal degradation of client proteins, oxidized and down-regulated in AD	[124, 125]
UBQLN1	UBQLN1 activity is necessary to regulate the production of APP and APP fragments	[127, 128]
mTOR	Inhibitory and/or activating modulation of mTOR through Aβ42 but not Aβ40	[143–146]
BECLIN1	BECLIN1 is down-regulated in AD brains and consequently increases APP levels and its metabolites	[161, 162]

*Aβ* amyloid beta, *APP* amyloid precursor protein, *HSF1* heat shock transcription factor 1, *GRP78* glucose-related protein 78, *HSP* heat shock protein, *CHIP* carboxy terminus of HSC70-interacting protein, *BAG1* BCL2-associated athanogene 1, *PS1* Presenilin 1, *UCH-L1* ubiquitin carboxy terminal hydrolase isozyme 1, *UBQLN1* Ubiquilin 1, *mTOR* mammalian target of rapamycin, *DUB* deubiquitination enzyme, *ER* endoplasmic reticulum

### Chaperones and AD

The two main chaperone scaffolds are HSP70 and HSP90 which are accompanied by a variety of co-chaperones specifying substrate binding and release and are transcriptionally regulated through heat shock elements (HSE) via the transcription factor HSF1. Interestingly, promoter studies of the *app* gene showed HSEs within its promoter region suggesting an impact of HSPs on AD pathology [81, 82]. As APP is a membrane-associated protein it maturates in the endoplasmic reticulum (ER) and the Golgi apparatus [83]. There, the ectodomain of APP associates with the luminal localized ER chaperone glucose-related protein 78 (GRP78, the ER isoform of HSP70) and this interaction impairs its maturation resulting in a reduced generation of Aβ40 and Aβ42 [84, 85]. The cytosolic HSP70 co-chaperone CHIP interacts with intracellular domain of APP in the ER and Golgi compartments providing a link between molecular chaperones and the UPS in APP processing [86]. In addition to the ER and Golgi-associated HSPs, the cytosolic chaperones are as well linked to APP and the Aβ metabolism [87]. The sHSPs HSP22 and HSP27 have been shown to bind to fibrillar Aβ to inhibit further fibrillarization. Moreover, HSP70 and HSP90 inhibit early stages of Aβ aggregation [88, 89]. HSPs have also been associated with extracellular Aβ as they can be released by either active secretion mechanisms or from cells undergoing necrosis [90, 91]. In AD brains, HSP90 co-localizes with amyloid plaques. Furthermore, extracellular HSP90 and HSP70 increased the amount of Aβ42 peptides in microglia after 1 day and decreased the amount after 3 days in vitro. These observations suggest that HSP-induced microglial activation may have a neuroprotective role by facilitating Aβ clearance [90].

Besides extracellular Aβ accumulation, the second major hallmark of AD is the intracellular aggregation of Tau. Post-translational modifications of Tau, e.g. hyper-phosphorylation, affect the conformation of the protein and promote the aggregation state. These post-translational modifications impact the interaction of Tau with microtubules, and thus, there may be specific forms of Tau that are preferred chaperone substrates relative to others. HSP27, HSP70 and CHIP are reported to recognize abnormal Tau and reduce its concentration by assisting its degradation and dephosphorylation [92–94]. HSP27 preferentially binds to hyper-phosphorylated but not to non-phosphorylated Tau and is cross-linked with Tau in NFTs from AD brains [94, 95]. Protein levels of soluble Tau positively correlate with HSP27, HSP40, HSP90, alphaB-crystallin and CHIP levels in AD brains [96]. Vice versa HSP protein levels are inversely correlated with levels of Tau oligomers, which are an intermediate of Tau filaments. In different cellular models, it has been shown that increased HSP70 and HSP90 levels promote Tau solubility and microtubule binding [92]. Tau seems to directly bind to HSP70 and this interaction is mediated by the HSP70 co-chaperone BAG1 pointing to a tight interplay between molecular chaperones and the UPS in counteracting Tau aggregation [97, 98]. Collectively, these data suggest that up-regulation of molecular chaperones may suppress formation of NTFs by partitioning Tau into a productive folding pathway and thereby preventing Tau aggregation.

### Ubiquitin–Proteasome System and AD

The ubiquitin–proteasome system is the main degradation pathway in the cell and it has been shown that Aβ40 and Aβ42 can block proteasome function in vitro. Aβ40 binds to the inner surface of proteasomes and inhibits 20S chymotrypsin-like activity. Aβ42 can inhibit proteasome function to an extent that is comparable to a well-known proteasome inhibitor [99–102]. It still has to be elucidated which form of Aβ is now affecting proteasome function. There is experimental evidence that Aβ oligomers but not monomers or fibrils impair long-term potentiation in vivo [103]. In a cell free proteasome activity assay, it has been shown that indeed Aβ40 and Aβ42 oligomers, but not monomers, decreased proteolytic activity of the proteasome in a dose-dependent manner [104]. The accumulation of Aβ is dependent on the balance between Aβ production and degradation. There is evidence suggesting that Aβ is degraded by the proteasome because inhibition of the proteasome with lactacystin caused a significant increase in Aβ42 levels in cultured neurons and astrocytes [101]. Consistent with these results, it has been shown that the 20S proteasome degrades Aβ40 and Aβ42 in vitro and that inhibition of the proteasome in cultured cells increases intracellular Aβ40 and Aβ42 level [104]. So far, it is still a matter of debate how the cytoplasmic and nuclear localized proteasome could physically interact with extracellular or luminal localized Aβ in vivo. In cultured neurons, it has been shown via immunoelectron microscopy that 20S proteasome subunits are detectable in the outer membranes and inner vesicles of multivesicular bodies [105]. In normal and AD brain, Aβ42 has also been shown to accumulate predominantly in multivesicular bodies, indicating that these structures are the possible interaction site of Aβ and the proteasome in vivo [106]. Further supporting the influence of the UPS on Aβ metabolism, it has been shown that PS1 and PS2 are degraded via the proteasome. Thus, a decrease in proteasome activity would increase γ-secretase activity and thereby Aβ production [107]. This illustrates a clear association between Aβ peptides and the proteostasis network, highlighting the importance of chaperones and the UPS in Aβ metabolism.

The protein Tau is degraded by the 26S and 20S proteasome in vitro and the use of proteasome inhibitors in cells and animal models leads to an accumulation of Tau [108–111]. Furthermore, mass spectrometry studies on isolated Tau from inclusion bodies show K48 and K63 ubiquitin linkages on Tau, which are the recognition signals for degradation via the UPS [112–114]. It has also been shown that the 20S proteasome co-precipitated with Tau aggregates and that the amount of pulled down Tau aggregates was higher in samples with low proteasome activity, suggesting an inhibitory interaction between Tau aggregates and proteasome activity [115]. In addition, in vitro studies show that Tau aggregates isolated from human AD brain directly inhibit the proteasome. In contrast, non-aggregated Tau isolated from AD brain or from control brain did not interfere with proteasome activity. These data show that different aggregation states of Tau modify its turnover via the proteasome [115]. Particularly intriguing is the aforementioned ubiquitination of Tau. It has been shown that Tau co-immunoprecipitates with CHIP, an E3 ubiquitin ligase, that ubiquitinates Tau for subsequent degradation via the proteasome and soluble phosphorylated Tau accumulates in brains of CHIP knockout mice [93, 116, 117]. In AD, the mechanism of stabilization and accumulation of hyper-phosphorylated Tau may involve inhibition of Tau interaction with CHIP [116]. In addition to phosphorylation, Tau is also post-translationally acetylated and this acetylation impairs proteosomal degradation and enhances accumulation of Tau [118]. Moreover, changes in the combination of proteasome subunits have been reported in AD brain, resulting in an altered proteasome activity [119]. Taken together, these data clearly indicate that proteasome activity is necessary for Tau turnover and that aggregated Tau inhibits proteasomal function.

Ubiquitin immunoreactivity accumulates in aggregates in AD brains and it has also been shown that some of these structures enclose ubiquitin-B mutant protein (UBB+1). Overexpression of this mutant, which contains a C-terminal amino acid extension, leads to an impairment of the proteasome and induces neuronal cell death [120, 121]. Oxidative stress is one factor that leads to protein damage and subsequently to protein ubiquitination and degradation via the proteasome [50]. It is interesting to note that the ubiquitin carboxy-terminal hydrolase L1 (UCH-L1) is oxidized in AD patients and is down-regulated in specific brain regions of early AD cases [122, 123]. UCH-L1 functions as a deubiquitination enzyme (DUB) that hydrolyses ubiquitin from polyubiquitinated proteins to liberate and stabilize ubiquitin monomers and it promotes proteasomal degradation. Interestingly, when overexpressed, UCH-L1 reverses behavioral deficits in AD model mice consistent with an impairment of the UPS in AD [124, 125]. Several lines of evidence implicate a tight regulation of the UPS and a strong interaction with autophagic pathways to maintain proteostasis. For example, Tau seems to be a substrate of the UPS and of the autophagic system in vivo. Recently, it has been shown that truncated Tau (TauΔc) is rapidly degraded via the autophagic system whereas non-truncated Tau is favorably degraded via the UPS [126]. In addition, Tau gets targeted for degradation through HSP70 regardless of its phosphorylation state involving the molecular chaperone machinery, which in turn interacts with the UPS-related proteins BAG1 and CHIP, both known to be involved in Tau degradation [97].

Taken together, these data indicate that decreased UPS function may be involved (or even partially causative) for AD pathogenesis. This view is further strengthened by recent genetic evidence showing a positive association between AD and several single nucleotide polymorphisms in an ubiquitin-like protein called ubiquilin 1 (UBQLN1). UBQLN1 is capable of preventing the aggregation of APP both in vitro and in vivo [127, 128]. Interestingly, mutations in another member of the ubiquilin family, ubiquilin 2 (UBQL2), are associated with the motor neuron disease ALS, which supports a general role of the UPS in neurodegeneration [129].

### Autophagy and AD

Emerging evidence suggests a contribution of autophagic pathways to AD pathogenesis. In fact, already in 1967 Suzuki and Terry reported dystrophic neurite swellings filled with vacuolar structures positive for acid phosphatase [130]. These structures have been shown to be unique for AD and ultrastructural imaging of AD brains revealed that they consist of lysosomes and a bulk of autophagic vacuoles (AVs) [131, 132]. These histological changes reflect either an increased synthesis of components of the lysosomal system, a disturbed clearance of AVs, or both, as the accumulation of vesicles can be initiated by blocking lysosomal activity in primary neuronal cell lines [133–135]. A general disturbance of neurite transport mechanism is not causative for the accumulation of AVs as the transport of organelles such as mitochondria appears unaltered under conditions of lysosomal inhibition [135]. Neuronal tissue might be extraordinarily susceptible for disturbances within the autophagic-lysosomal system, because this network enables neurons to perform highly specific functions such as vesicle release and synaptic transmission [136]. Furthermore, post-mitotic neurons have to cope with elevated levels of stress and damaged organelles as well as misfolded or aggregated proteins during their lifetime, cellular aging and disease progression, which highlight the importance of an effective degradation system in this particular cell type [137].

Besides changes of the autophagic system on a histological level, also molecular regulators of autophagic degradation are altered during AD progression. Genes that represent negative regulators of autophagy are repressed, whereas positive modulators are more likely to be up-regulated in the brain of AD patients [138]. However, enhanced expression of autophagy activators does not result in a persistent increase of autophagic activity as the autophagic efficacy declines during the course of the disease, resulting in an accumulation of undegraded proteins [139]. A key regulator of autophagic activity is mTOR. The most prominent physiological modulator of mTOR signaling is caloric restriction (CR) meaning the energy content of food is reduced without compromising the supply of essential nutrients. CR represses mTOR signaling and therefore enhances autophagic activity [140]. Attenuation of AD as well as a prolonged lifespan in healthy humans mediated by CR might not only be due to reduced metabolic toxicity and decreased vulnerability to metabolic diseases like diabetes, but may also rely on cytoprotective effects of mTOR-mediated autophagic activity [141, 142]. Interestingly, Aβ42, but not Aβ40, affects mTOR signaling and is as well affected by mTOR modulation itself. The discussion, if Aβ silences or enhances mTOR signaling is still ongoing: some studies show inhibitory effects of Aβ on mTOR signaling, while others show an activation [143–146]. In a murine neuroblastoma cell line (N2a) and hippocampal slice cultures exposed to Aβ42, in APPswe/PS1, a double-transgenic mouse model for AD, and in lymphocytes of AD patients, the phosphorylation of mTOR as well as the phosphorylation of its substrate p70S6-kinase is reduced [144, 145]. Furthermore, in the Tg2576 APP-overexpressing mice, the Aβ-mediated reduction of mTOR signaling has been shown to be accompanied by a decline in synaptic activity that can be rescued by mTOR up-regulation [145]. In contrast, cells which produce high levels of Aβ oligomers show an enhanced mTOR activity which can be counteracted by decreasing Aβ formation or administration of rapamycin. Similar results have been seen in triple transgenic AD mice (3×Tg-AD mice), combining Aβ and Tau-pathology of AD, which show a reduction in Tau phosphorylation and improved learning and memory after rapamycin treatment. Interestingly, the effects on mTOR signaling are only observed in brain areas such as hippocampus and cortex which are also affected in brains of AD patients, but not in others [143]. The beneficial effect of rapamycin treatment and, therefore, autophagy stimulation on learning and memory is also reported for a further AD mouse model. These animals show a recovery of the autophagic flux by enhanced autophagic activity in the hippocampus after administration of rapamycin [146].

Interestingly, already in 1992, Haass and colleagues reported a lysosomal pathway that is involved in APP processing and potentially responsible for the generation of amyloid-bearing fragments in AD via the generation of an extensive array of APP C-terminal fragments (APP-CTFs) which are found in lysosomes [147]. Later, alterations in the metabolism of APP have been shown in models for glycosphingolipid storage disease, where a mTOR-independent increase in the autophagic vacuole-associated protein LC3-II occurs, indicating an impaired lysosomal flux. This suggests an anti-amyloidogenic role of lysosomal proteolysis in post-secretase APP-CTF catabolism, which does not directly involve macroautophagy [148]. The accumulation of sphingolipids, which characterizes lysosomal lipid storage disorders, has also been shown to decrease the lysosome-dependent degradation of APP-CTFs and to stimulate γ-secretase activity [149]. Thus, sphingolipids might trigger increased generation of Aβ via impairment of the autophagic lysosomal system and contribute to neurodegeneration in sporadic AD [149]. Pharmacological enhancement of autophagy or induction of autophagy via starvation greatly decreased the levels of Aβ peptides and APP-CTFs in a γ-secretase independent manner. Consequently, after inhibition of autophagy, a significant accumulation of Aβ peptides and APP-CTFs has been observed [150]. The upcoming data support the involvement of autophagy in the clearance of Aβ and APP-CTFs.

The autophagy receptor p62/SQSTM1 shows a reduced expression in AD patients, as well as in 3×Tg-AD-expressing mice [151]. This might be caused by oxidative stress and subsequent DNA damage of the *p62/sqstm1* promoter, which leads to reduced *p62/sqstm1* transcription [151, 152]. Because guanine is the nucleobase which is most susceptible to oxidation, the *p62/sqstm1* promoter is exceptionally prone to oxidative stress, as it is rich of GC regions [153, 154]. Interestingly, Tau is degraded via the autophagic-lysosomal system, whereby Tau aggregates are cleared via macroautophagy [155]. As Tau possesses two putative CMA-targeting motifs, soluble Tau is most likely degraded via CMA [155]. Hyper-phosphorylated Tau accumulates in *p62/sqstm1*-knockout mice [156], indicating an interplay of oxidative stress and the clearance of protein aggregates in AD. Another important modulator of autophagic activity is BECLIN 1 which is a hub-protein-forming multiprotein complexes that possess different functions according to their composition [157–160]. BECLIN 1 is down-regulated in the brain of AD patients even in cases of mild cognitive impairment. In addition, mutant APP-overexpressing mice with a heterozygous deletion of BECLIN 1 show enhanced microglial activation and an accumulation of lysosomes [161]. Moreover, the knockdown of BECLIN 1 in cultured cells increases the levels of APP and its cleavage products which are accompanied by impaired autophagic flux. However, the overexpression of mutant APP has no effect on BECLIN 1 expression in cells and mice [161, 162].

Recently, it has been shown that PS1 is important for a normal autophagic-lysosomal function independent of Aβ [163]. PS1 has been found to be important for the acidification of the lysosomal compartment and thus to be crucial for lysosomal proteolysis and autophagic function. In neurons lacking PS1, the subunit V01A of the vacuolar ATPase is not correctly delivered to the lysosomes and not properly integrated into the lysosomal membrane. This subunit is needed for correct function of the proton pump, which is responsible for lysosomal acidification. γ-secretase lacking PS1 is not able to sufficiently facilitate N-glycosylation of the V01A subunit in the ER, which is crucial for efficient transport to the lysosomes and assembly of the functional proton pump. As a result, V01A is entrapped in the ER and cells loose lysosomal function. In addition, an accumulation of AVs is seen in PS1 hypomorphic mice and the pharmacological block of lysosomal acidification leads to similar accumulation of lysosomes and AVs in primary neuronal cell lines [163]. The first abnormalities of the lysosomal-endosomal system occur prior to other hallmarks of the disease such as amyloid deposits in the neocortex and persist until massive impairments of the autophagic system emerge in the later stages of the disease [164]. Taken together, all these findings suggest an important role for autophagy in the pathogenesis of AD.

## Outlook

There is quite some evidence that in AD, the proteostasis network is impaired. Based on pathological analysis, AD mouse models, in vitro and cellular investigations a molecular link between chaperones, the ubiquitin–proteasome system, autophagy pathways and pathogenetic mechanisms of AD can be suggested. Deregulation and changes of chaperones and proteasome activity might have serious implications for aging as well as for age-associated diseases. Autophagy pathways are key mechanisms and of vital importance for cellular function and, especially, for cell survival under adverse conditions. Consequently, an effect of proteostasis control on neurodegeneration or, vice versa, of neurodegeneration on proteostasis is reasonable. As with other pathomechanisms that are investigated in the search for the cause of AD, it is still open whether an impairment of proteostasis is an upstream or downstream event during AD onset and progression. Experimental approaches employing mouse models clearly demonstrate that stabilization or induction of proteostasis can be neuroprotective. Whether this can be translated into the human condition and, most importantly, whether supporting proteome integrity can be a real target for pharmacological intervention for the prevention and treatment of AD is currently still open. Also, one has to consider that, e.g. the stimulation of general autophagy may in the long term lead to the stimulation of proliferation of non-neuronal cells, eventually resulting in tumor formation. Indeed, a permanent autophagy induction is known as one cellular mechanism that promotes the escape of cells from proliferation control. As beneficial it may be to support autophagy in post-mitotic neurons that are confronted with disturbed proteostasis and an impairment of proteasome function, this stimulation should target specific autophagy pathways, such as selective chaperone-mediated macroautophagy involved in the degradation of disease-associated protein aggregation. Although there are obviously still many questions to be answered to understand the role of proteostasis in AD (and also in other age-associated neurodegenerative disorders), it is a big step forward in AD research to consider a possible role of pathogenetic pathways that are not directly linked to the usual suspects Aβ and Tau. A better understanding of how the proteostasis network is regulated might help to identify targets which lead to prevention of the deleterious loss of neuronal cells and tissue in AD.
